# Endothelin receptor antagonist improves donor lung function in an ex vivo perfusion system

**DOI:** 10.1186/s12929-020-00690-7

**Published:** 2020-10-02

**Authors:** K. Walweel, K. Skeggs, A. C. Boon, L. E. See Hoe, M. Bouquet, N. G. Obonyo, S. E. Pedersen, S. D. Diab, M. R. Passmore, K. Hyslop, E. S. Wood, J. Reid, S. M. Colombo, N. J. Bartnikowski, M. A. Wells, D. Black, L. P. Pimenta, A. K. Stevenson, K. Bisht, L. Marshall, D. A. Prabhu, L. James, D. G. Platts, P. S. Macdonald, D. C. McGiffin, J. Y. Suen, J. F. Fraser

**Affiliations:** 1 Critical Care Research Group, Level 3, Clinical Sciences Building, The Prince Charles Hospital, Rode Road, Brisbane, Australia; 2 Princess Alexandra Hospital, Woolloongabba, Brisbane, QLD 4102 Australia; 3 Initiative to Develop African Research Leaders, KEMRI-Wellcome, Trust Research Programme, Kilifi, Kenya; 4 University of Milan, Milan, Italy; 5 Queensland University of Technology, Brisbane, Australia; 6 School of Medical Science, Griffith University, Brisbane, Australia; 7 Mater Research Institute-The University of Queensland, Woolloongabba, QLD Australia; 8 The Prince Charles Hospital, Rode Road, Brisbane, Australia; 9 Cardiac Mechanics Research Laboratory, St. Vincent’s Hospital and the Victor Chang Cardiac Research Institute, Victoria Street, Darlinghurst, Sydney, NSW 2061 Australia; 10 Cardiothoracic Surgery and Transplantation, The Alfred Hospital, Melbourne, Australia

**Keywords:** Brain stem death, Lung transplantation, EVLP, Endothelin axis, Tezosentan

## Abstract

**Background:**

A lung transplant is the last resort treatment for many patients with advanced lung disease. The majority of donated lungs come from donors following brain death (BD). The endothelin axis is upregulated in the blood and lung of the donor after BD resulting in systemic inflammation, lung damage and poor lung graft outcomes in the recipient. Tezosentan (endothelin receptor blocker) improves the pulmonary haemodynamic profile; however, it induces adverse effects on other organs at high doses. Application of ex vivo lung perfusion (EVLP) allows the development of organ-specific hormone resuscitation, to maximise and optimise the donor pool. Therefore, we investigate whether the combination of EVLP and tezosentan administration could improve the quality of donor lungs in a clinically relevant 6-h ovine model of brain stem death (BSD).

**Methods:**

After 6 h of BSD, lungs obtained from 12 sheep were divided into two groups, control and tezosentan-treated group, and cannulated for EVLP. The lungs were monitored for 6 h and lung perfusate and tissue samples were processed and analysed. Blood gas variables were measured in perfusate samples as well as total proteins and pro-inflammatory biomarkers, IL-6 and IL-8. Lung tissues were collected at the end of EVLP experiments for histology analysis and wet-dry weight ratio (a measure of oedema).

**Results:**

Our results showed a significant improvement in gas exchange [elevated partial pressure of oxygen (P = 0.02) and reduced partial pressure of carbon dioxide (P = 0.03)] in tezosentan-treated lungs compared to controls. However, the lungs hematoxylin–eosin staining histology results showed minimum lung injuries and there was no difference between both control and tezosentan-treated lungs. Similarly, IL-6 and IL-8 levels in lung perfusate showed no difference between control and tezosentan-treated lungs throughout the EVLP. Histological and tissue analysis showed a non-significant reduction in wet/dry weight ratio in tezosentan-treated lung tissues (P = 0.09) when compared to control.

**Conclusions:**

These data indicate that administration of tezosentan could improve pulmonary gas exchange during EVLP.

## Introduction

Lung transplantation is the ultimate solution for patients with end stage respiratory failure; however, its success is limited by significant donor organ shortages [1–6]. Lungs donated for transplantation are primarily sourced from brain dead organ donors. However, brain death (BD) is associated with systemic inflammation, haemodynamic and endocrine effects that lead to pulmonary complications [7, 8]. BD induces lung injury via release of diverse growth factors and inflammatory mediators that act as stimuli for a systemic inflammatory cascade [9–12]. Additionally, the process of BD itself may not only damage the lung directly but also jeopardize its function post-transplantation [10, 13]. However, the pathophysiologic mechanisms of BD-induced lung functions are not fully elucidated.

Recent findings suggested that activation/dysfunction of the pulmonary endothelium is critical for BD-induced lung injuries [7, 9]. The endothelial dysfunction is manifested by activation of a number of endothelial biomarkers (endothelin, cell adhesion molecules and selectins) [14, 15], which could lead to reduced graft survival after BD [16, 17]. Endothelins (ET) are a family of 21 amino acid peptides and exist in three isoforms: ET-1, ET-2 and ET-3 [18, 19]. ET-1 is the most abundant isoform, which acts as a potent vasoconstrictor, smooth muscle cell and fibroblasts mitogen and a stimulator of inflammatory cell infiltration [18, 19]. Moreover, ET-1 increases the expression of cell adhesion molecules, indicating a link between ET-1 and endothelial dysfunction [20]. ET-1 mediates its effects via two distinct receptors: ET-A and ET-B [19]. ET-A receptors in the normal lung are expressed in vascular and airway smooth muscle cells, whereas ET-B receptors are abundant in endothelial cells [19]. Activation of ET-A and ET-B receptors promotes vasoconstriction and bronchoconstriction, respectively [19]. The endothelin axis (endothelins, their precursors, receptors and associated signalling pathways [21, 22]) stimulates matrix metalloproteinase expression in pulmonary tissue, resulting in protein hydrolysis and interstitial oedema [21]. Because ET-1 may act as an immune modulator, an increase in ET-1 may contribute to lung injuries by inducing the expression of cytokines, including IL-6 and IL-8 [23]. We have previously shown that the pulmonary endothelin axis is upregulated in the blood circulation and donor's lung after brain stem death (BSD) [21, 22]. Interestingly, blockade of ET receptors has been reported to improve vascular function and pulmonary arterial hypertension in various animal studies [24–26].

Tezosentan, a dual endothelin antagonist, is a novel compound with a rapid onset of action in several animal models of heart failure, ischemic renal failure, and hypertension [27]. Tezosentan competitively antagonizes the specific binding of ET-1 and ET-3 on cells and tissues carrying ET-A and ET-B receptors, with inhibitory constants in the nanomolar range [24, 25]. It is water-soluble, thus allowing its administration both intravenously and via nebulisation [24, 27, 28]. It has been effective in reducing pulmonary hypertension and pulmonary vascular resistance in several animal models of induced lung injury [24, 26, 28–32]; however, tezosentan has acute hemodynamic effects such as a decrease in blood pressure [24, 28]. Moreover, tezosentan induces adverse effects on other organs at high doses [33].

A key approach in lung transplantation is the introduction of ex vivo lung perfusion (EVLP), a novel strategy to overcome the shortage of available donor lungs [34–37]. EVLP allows evaluation and reconditioning of lungs outside the donor, providing an opportunity to improve lung function before transplantation [35, 36, 38]. EVLP perfusate, a sampling source to assess the lung during EVLP, offers valuable information about the condition of the donor lung [39–43]. Detection of lung injury markers in the perfusate may also help finding future targeted treatments that could be administered directly to the lungs through the EVLP circuit [44]. Several studies have reported the use of the EVLP system as a device for direct pharmacologic graft intervention in large-animal models [45–48] and in patients [49, 50]. In line with this concept, we are reporting the application of EVLP with tezosentan to understand the pathophysiology of the ET system and reveal the impact of tezosentan on reversing the endothelial dysfunction lung injury in our established BSD-induced ovine model. The combination of tezosentan with the EVLP allows the use of greater doses of the drug (10 mg/kg) administered directly to the target organ and avoids systemic adverse effects in the donor at the same time. We found that tezosentan administration resulted in improved pulmonary gas exchange post BSD with improved oxygenation in the lungs during EVLP.

## Methodology

### Animal BSD model

Twelve female merino sheep (37‒42 kg, 2 years old) underwent BSD procedures for 6 h as previously developed and described by our group [22]. The animals only had access to drinking water during the night prior to the experiment. General anaesthesia was induced with midazolam (0.5 mg/kg) and ketamine (5 mg/kg), and animals were intubated under direct laryngoscopy. Following anaesthetic induction, all animals were mechanically ventilated and standard instrumentation procedures were performed [51]. Briefly, a cranial burr hole was created midway between the midline and lateral edge of the cranium followed by the extradural placement of 5.3 mm Foley catheter (Brad BIOCATH, United Kingdom). One hour after completion of all invasive procedures, BSD was induced by slowly inflating the intracranial catheter with 30 mL saline over 30 min to increase intracranial pressure (ICP) above the mean arterial pressure (MAP). Confirmation of BSD was achieved by continuously negative cerebral perfusion pressure (defined as MAP-ICP) for greater than 30 min, loss of pupillary and corneal reflexes and lack of respiratory efforts. All sheep received hormone resuscitation 3 h following confirmation of BSD: triiodothyronine (4 μg bolus and 3 μg/h infusion), vasopressin (1 unit bolus followed by 0.5–4.0 U/h infusion, adjusted to SVR 800–1200 dyn s/cm^5^) and methylprednisolone (15 mg/kg) [22]. Sheep were monitored and hemodynamically managed for 6 h after BSD confirmation, then humanely sacrificed with sodium pentobarbitone (100 mg/kg). All animal experiments were performed at the Medical Engineering Research Facility (Queensland University of Technology; QUT) and approved by the QUT Animal Ethics Research Committee.

### Administration of tezosentan

Atalay et al. have used 10 mg/kg of tezosentan to attenuate lung injury in alpha-naphthylthiourea-induced acute lung injury in rats [52]. A single dose of 400 mg of tezosentan (Actelion Pharmaceutical, Switzerland. 10 mg/kg) was added to the prime solution prior to initiation of EVLP based upon the average expected sheep weight of 40 kg (reconstituted to 5 mL with normal saline). Tezosentan was administered as an infusion throughout all 6 h of EVLP at a constant rate of 0.5 L/min. Because tezosentan is cleared by liver and kidney, we anticipated that the half-life would be longer during EVLP and an indefinite duration of action [53]. In cases where the drug could not sustain the full 6 h of EVLP, data points recorded in the next hours after the premature end of EVLP were considered the same as the last data point available to allow comparison at all evaluation points. Therefore, at the end of EVLP the last available data point is included for the statistical analysis.

### Study protocol

After 6 h of BSD, the lungs were retrieved from BSD sheep as previously described [54]. Lungs were flushed with 1 L of organ preservation solution (Perfadex, XVIVO Perfusion, Uppsala, Sweden) at 4 °C through the pulmonary artery (PA) cannula. Ventilation was continued throughout the extraction of the lung block. The trachea was clamped with the lungs inflated with a sustained airway pressure and the lungs were immersed in Perfadex until EVLP. The study consisted of control and tezosentan-treated groups (n = 6 each). Saline was given to the control group and 10 mg/kg tezosentan was administered to the treatment group, which were added to the prime solution.

### EVLP technique

**Fig. 1:**
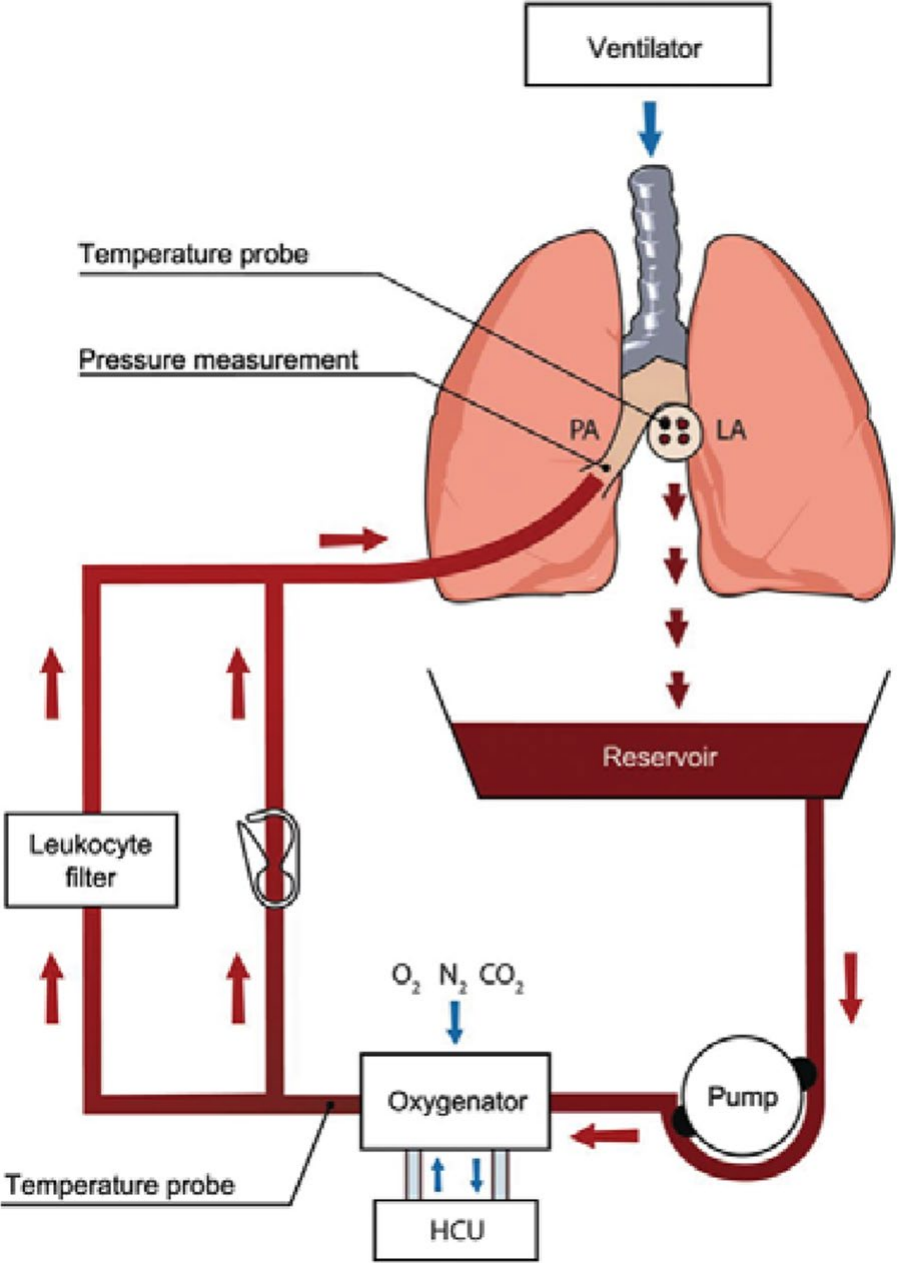
Schematic diagram of an EVLP circuit (Vivoline LS1 system, Vivoline Medical AB, Lund, Sweden). *HCU* heater cooler unit, *LA* left atrium, *PA* pulmonary artery [55]

The EVLP system consists of a perfusion circuit with oxygenator, leukocyte filter and a reservoir according to manufacturer’s instruction (Vivoline, offered by XVIVO Lung Perfusion Sweden, Fig. 1). The circuit was primed with 1.5 L of Steen solution (XVIVO Perfusion) and warmed to 32 °C. Sodium heparin (10,000 IU), 500 mg of methylprednisolone, 500 mg cefazolin and 3 units of packed red blood cells (blood cross-matching was carried out prior to this stage) were added to the perfusate. Perfusate was pump driven from a reservoir through a gas exchange membrane, heat exchanger, and leukocyte filter before entering the lungs via the PA (Fig. 1). Pulmonary effluent from left atrium (LA) drains back to the reservoir and is recirculated. The oxygenator was used to deoxygenate the perfusate using a gas mixture (8% CO_2_, 6% O_2_, 86% N_2_). During this phase, the operation of the system was commenced with a flow of 0.5 L/min set to a maximum of 2 L/min and an initial maximum pressure of 10 mmHg. The priming mode was run for 15 min before the lungs connect to allow adequate mixing of the solution with gases. Sample of the perfusate was drawn for biochemical analysis to correct pH, HCO^−^_3_ and glucose levels as needed. Lungs were then placed within the EVLP chamber, degassed, and low flow (0.5 L/min) through the lungs was established to initiate EVLP. Upon initiation of perfusion with careful monitoring of PA pressure maintained from 15 to 20 mmHg and maximum flow circulation of 4 L/min (recommended maximum circulation of 100 mL/kg/min). Once the temperature of the outflowing perfusate has reached 32–34 °C, protective lung ventilation was started (tidal volume 10 mL/kg donor weight; respiratory rate 12 breaths per minute; positive end-expiratory pressure (PEEP) 10 cm H_2_O and FiO_2_ 100%). Lung temperature was allowed to increase to 38 °C after which saline (control) or tezosentan (treatment group) were administered. EVLP was then performed for 6 h.

### EVLP assessment and parameters

The graft perfusate samples in both control and tezosentan-treated groups were evaluated and examined during EVLP for gas exchange variables, protein concentration (pulmonary oedema) and inflammatory biomarkers, IL-6 and IL-8. Lung perfusate samples were collected in the effluent and affluent arms of the EVLP circuit to measure PO_2_ and PCO_2_. The difference between PO_2_ in both arms was calculated as the oxygenation capacity (ΔpO_2_) of the lungs. ΔpCO_2_ was calculated as the difference in CO_2_ partial pressure in LA before and after reperfusion. Most important parameters monitored during assessment are listed in Table 1.

**Table 1:** Base line parameters of the donor lungs

	Control	Tezosentan	P values
pH	7.4 ± 0.05	7.5 ± 0.08	0.32
Beecf (mmol/L)	1.3 ± 1.0	1.6 ± 2	0.22
HCO_3_ (mmol/L)	25 ± 0.8	22 ± 3.4	0.25
TCO_2_	26 ± 0.8	22 ± 3.6	0.28
SO_2_%	100 ± 0	100 ± 0.0	**–**
Lactate (mmol/L)	2.1 ± 0.3	2.7 ± 1.2	0.58
WBC ×109/L	0.93 ± 0.3	1.7 ± 0.4	0.15
RBC ×109/L	2.8 ± 0.15	3.1 ± 0.2	0.40
Hb g/L	32 ± 2.0	36 ± 3.0	0.30
Htc %	0.09 ± 0.01	0.1 ± 0.01	0.30

Values are mean ± SEM. P values are for control vs tezosentan

Pulmonary vascular resistance (PVR, Table 1) was reported as dynes s/cm^5^ and calculated as ((PA pressure − LA pressure) * 80/perfusion flow). Total protein concentrations in the perfusate samples (from reservoir) were used as a marker of permeability lung oedema. Protein quantification was performed using Coomassie Plus (Bradford) assay kit. Bovine serum albumin was used as a standard. Absorbance of standards and samples were determined spectrophotometrically using a microplate reader. Results were plotted against the linear portion of the standard curve, and the protein concentration of each sample was expressed as mg/L of sample. Lung perfusate samples were also assayed to determine the release of cytokines, IL-6 and IL-8. The perfusate samples were centrifuged at 1800 rpm for 8 min and the supernatant was then stored at – 80 °C until analysis. Quantification of cytokine levels was assessed using a commercially available sheep cytokine multiplex immunoassay kit plate reader (Abacus, Australia) and the concentration was expressed as pg/mL.

### Tissue sampling

At the end of EVLP experiment, tissue samples were taken for histological evaluation and wet-to-dry weight ratio calculation. To assess lung injury, tissue samples were collected, fixed in formalin and embedded in paraffin. Tissue sections were stained with haematoxylin and eosin for microscopic assessment. Wet-to-dry weight ratios were determined as an additional measure of pulmonary oedema. The wet-to-dry weight ratio for each group (control or treated) was calculated as the mean of the ratios from all 6 lungs tissue samples. It was measured by weighing the tissue samples immediately at the end of EVLP (wet weight). This tissue was then placed in an Eppendorf tube, which was left open at room temperature for a minimum of 2 weeks. Once the tissue desiccated, it was weighed again (dry weight). A wet to dry lung weight ratio was then calculated and compared between the two groups.

### Statistics

Group data were presented as the mean ± standard error of the mean (SEM) and analysed as a time series. The statistical analysis was performed using Graphpad Prism 6 software using non-parametric t-test (Mann–Whiteney test) and the level of significance was set at P < 0.05.

## Results

### Lung function during EVLP

**Fig. 2:**
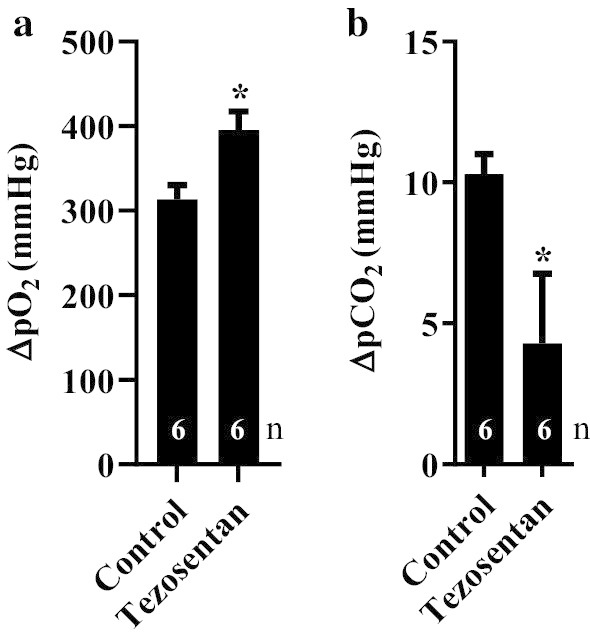
Gas exchange variables in BSD lungs treated with tezosentan compared to controls during EVLP. The oxygenation capacity (ΔpO_2_, **a**) was calculated as the difference in the partial pressure of O_2_ between the oxygenator (venous blood) and LA during evaluation phase. ΔpCO_2_ (**b**) was calculated as the difference in CO_2_ partial pressure in LA before and after EVLP. Data is expressed as mean ± SEM. *P < 0.05 versus control

Pulmonary gas exchange was significantly better throughout EVLP in tezosentan-treated group than in control group (Fig. 2). Oxygenation capacity (ΔpO_2,_ Fig. 2a) of the lungs was calculated as the difference in O_2_ partial pressure in LA before (treated, 48.2 ± 2.4; vs control, 52.2 ± 1.6; P = 0.2) and after (treated, 443.2 ± 24.6; vs control, 365.8 ± 16.9; P = 0.03) reperfusion. ΔpO_2_ was significantly greater in treated group (treated, 395 ± 22; vs control, 314 ± 17; P = 0.02, Fig. 2a). The difference in the partial pressure of CO_2_ (ΔpCO_2_, Fig. 2b) was calculated as the difference in CO_2_ partial pressure in LA before (treated, 26.4 ± 5.3; vs control, 27.9 ± 1.5; P = 0.78) and after (treated, 30.7 ± 6.4; vs control, 38.2 ± 1.7; P = 0.25) reperfusion. ΔpCO_2_ was significantly reduced in lungs treated with tezosentan compared to controls (treated, 4.3 ± 2.5; vs control, 10.3 ± 0.7; P = 0.03, Fig. 2b).

### Assessment of pulmonary oedema

**Fig. 3:**
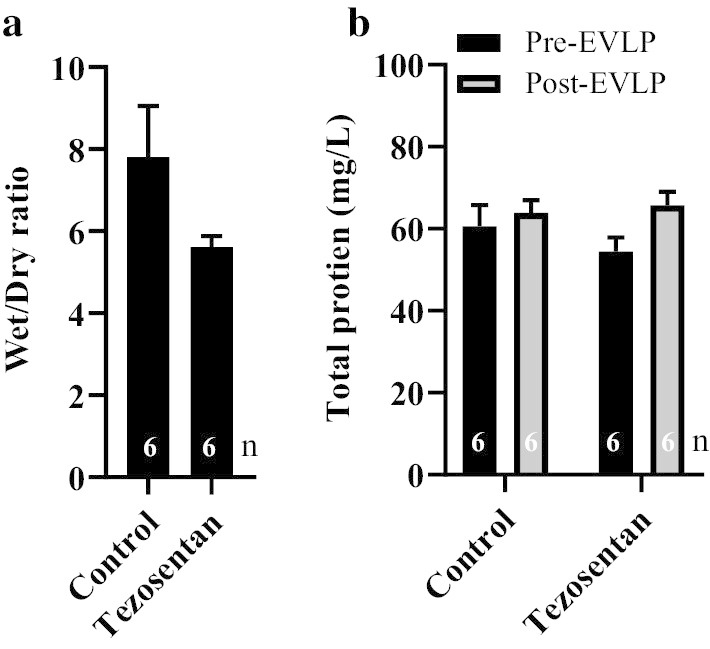
Indices of lung oedema. Lung wet-to-dry weight ratio (**a**) and total protein concentration (**b**) in tezosentan-treated lungs perfusate compared to controls. Wet-to-dry weight ratios were measured at the end of EVLP, whereas, total protein in perfusate samples were measured throughout the EVLP. Data is expressed as mean ± SEM

Lung wet-to-dry weight ratio was used as a measure of pulmonary oedema (Fig. 3a). Over the course of the 6-h EVLP, wet-to-dry weight ratio was lower in the group that received tezosentan compared with the control group, but the differences did not reach statistical significance (P = 0.09). We also measured the concentration of total protein in lung perfusate samples, as an index of permeability pulmonary oedema, before and after reperfusion (Fig. 3b). No change was detected in the total protein concentration in tezosentan-treated and control perfusate samples throughout EVLP.

### Pro-inflammatory cytokines

**Fig. 4:**
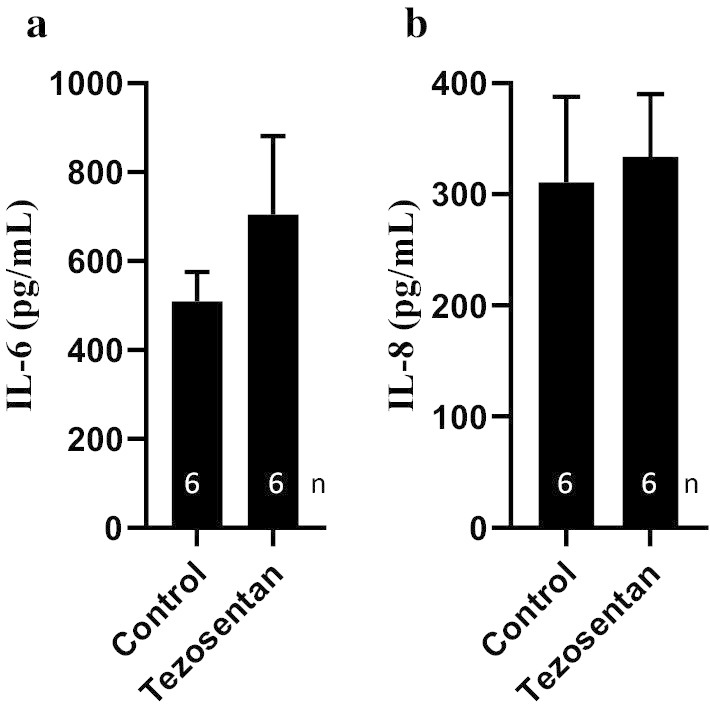
IL-6 (**a**) and IL-8 (**b**) levels in lung perfusate treated with tezosentan compared to controls during EVLP. Data is expressed as mean ± SEM

Evidence suggests that early endothelin release possibly contributes to the previously recognised pulmonary inflammation in potential donors [22]. To investigate whether the endothelin receptor blocker, tezosentan, has an effect on cytokines release in the lung, the concentrations of the pro-inflammatory cytokines, IL-6 and IL-8, were measured in the control and tezosentan-treated lung perfusate during EVLP (Fig. 4). Multiplex analysis showed no difference in the levels of IL-6 (Fig. 4a, treated, 509 ± 66; vs control, 704 ± 177; P = 0.33) and IL-8 (Fig. 4b, treated, 311 ± 77; vs control, 334 ± 56; P = 0.82) between the two groups throughout the EVLP experiment.

### Histology

**Fig. 5:**
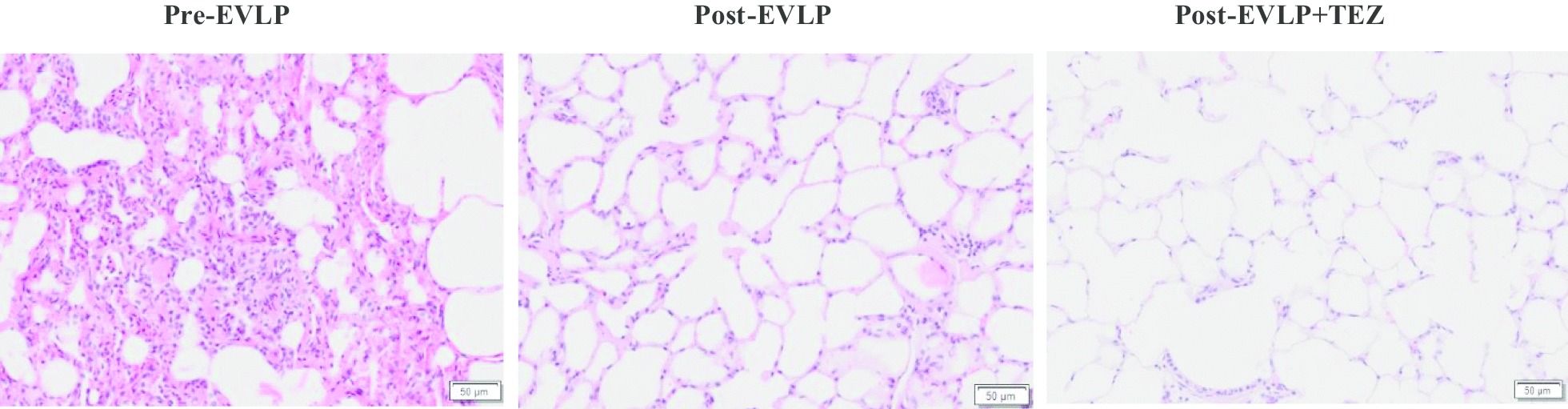
Microscopic BSD lung injuries before and after EVLP. Representative haematoxylin–eosin tissue sections of lungs before (Pre-EVLP) and after 12 h of reperfusion in presence and absence of tezosentan (Post-EVLP and Post-EVLP + TEZ). Representative sections are shown at ×40 magnification. *TEZ* tezosentan

Evaluation of haematoxylin and eosin-stained tissue sections showed minimum lung injuries (less inflammatory cell infiltration and alveolar haemorrhage) at the end of EVLP. However, tezosentan-treated groups showed less inflammatory cell infiltration and alveolar haemorrhage (Fig. 5).

## Discussion

The main findings of the current study are that the endothelin receptor antagonist tezosentan, administered during EVLP of sheep lungs, significantly reduced physiological deterioration after BSD. These findings indicate that pharmacological interference with the pro-inflammatory response, in combination with EVLP, may represent a useful option for the treatment of damaged lung grafts.

The effect of tezosentan on pulmonary haemodynamic profile was evident after only 30 min and was statistically significant at the 6-h end point of EVLP. Its clearance seems to be mostly hepatic and some renal elimination of unchanged drugs as there does not seem to be any meaningful metabolism and so a bolus into the EVLP circuit would be expected to have no elimination and an indefinite duration of action [53]. Administration of tezosentan by perfusate delivery improved the pulmonary oxygenation during EVLP in our established BSD-induced ovine model (Fig. 2). Our data are consistent with previous findings that show the beneficial effects of tezosentan administration on lung function [24, 26, 28–32]. Mommerot and his colleagues [28] observed improved hemodynamics and oxygenation parameters in a porcine model of cardiopulmonary bypass upon tezosentan administration. Similarly, Rossi et al. [32] have shown that tezosentan improves gas exchange in endotoxin-induced lung injury in pigs. Tezosentan was also able to reduce pulmonary hypertension in endotoxemic pigs [24], endotoxemic sheep [30] and in lambs with acute and chronic pulmonary hypertension [31]. Moreover, tezosentan decreased pulmonary vascular resistance and bronchiolar obstruction in sheep after smoke inhalation and burn injury [29]. These findings suggest that ET-1 is a mechanism for the protective effect of tezosentan in all these animals [40, 56–58]. ET receptors are present in both arterial and venous vessels, and the hemodynamic responses to tezosentan suggest that it blocks these receptors in both arteries and veins [59]. Mechanistic studies have shown that ET-1 promotes harmful cross talk between the endothelial and alveolar compartments by stimulating nitric oxide production, leading to impairment in alveolar fluid clearance and pulmonary oedema [40, 60]. In addition, ET-1 increases capillary hydrostatic pressure; induces inflammatory cells recruitment, which disrupts the endothelial/epithelial barrier; and upregulates mediators that increase vascular permeability [40, 60]. A recent study revealed that the oedema-promoting effects of ET-1 might be related to increased level of heparin-binding protein (HBP, released from neutrophils), which induces vascular hyperpermeability and contributes to oedema formation in the endotoxemic pig model [61]. Tezosentan‐treatment markedly attenuated plasma HBP and extravascular lung water in this model [61]. These findings indicate that tezosentan could be a potential therapeutic option to reduce lung injury via decreasing the permeability of the endothelial and epithelial barrier [56]. However, our data failed to detect pulmonary oedema in tezosentan-treated lungs compared to controls, as measured by wet-to-dry weight ratios. Our data is consistent with previous findings that showed tezosentan had no significant preventive effect on pulmonary oedema in the rat model of alpha-naphthylthiourea-induced acute lung injury [52]. This discrepancy may be due to different animal models of induced lung injury, ventilation, haemodynamic management/optimisation and hormone resuscitation which can ultimately affect the temporal inflammatory profile [62, 63]. Further studies are required to describe the specific mechanisms behind the favourable effects of ET receptor antagonism, tezosentan.

The cytokine expression in control and tezosentan-treated lungs was represented by evaluating cytokines in perfusate sample throughout the EVLP (Fig. 4). Administration of tezosentan had no influence on the levels of IL-6 and IL-8 in lung perfusate. Our data is consistent with previous findings that showed unchanged expression levels of IL-6, TNF-α or IL-10 in plasma samples obtained from endotoxemic pigs upon tezosentan administration [64]. However, Kuklin et al. [30] using endotoxemic sheep, have shown that plasma concentration of IL-8 and TNF-α were significantly higher in tezosentan-treated animals. The differences in the results we observed may be because cytokines expression during EVLP does not completely reflect the in vivo reperfusion situation [65] and its role on EVLP is still largely unknown [66]. Although tezosentan did not reduce the levels of pro-inflammatory cytokines in our study, there is no evidence of histologic injury resulting from reperfusion (Fig. 5). It has been shown that reduction of cytokines in lung perfusate did not affect oxygenation, PVR, or oedema formation, demonstrating that factors other than cytokines play a significant role in graft dysfunction [67]. In addition, EVLP on its own appears to have a positive influence on the injured lungs, which may be related to the optimal oncotic pressure of the perfusion solution [68]. Future studies are clearly needed to investigate cytokines expression during EVLP in presence of tezosentan and how this combination reduces organ inflammation.

## Study limitations

Several important limitations have been observed in the current study. Firstly, the effect of tezosentan on the expression level of oedema‐promoting protein HBP in sheep lungs obtained after BSD was not measured. Disruption of ET‐signalling in endotoxemia has been shown to attenuate formation of oedema via decreasing HBP levels [61]. Another limitation was related to physiological assessments. We observed significantly better oxygenation results in the treatment group during the reperfusion period; however, lung compliance was not measured. It has previously been advocated that compliance is the best parameter to predict donor lung quality [69, 70]. Finally, the expression of cytokine and inflammatory cells in lungs of both groups were represented by evaluating them in lung perfusate but not in the bronchoalveolar lavage, which could be different [65].

## Conclusion

Our study indicates that the endothelin receptor antagonist tezosentan, administered during ex-vivo perfusion of injured sheep lungs obtained after BSD, markedly alleviates physiological deterioration. Therefore, pharmacological therapy with endothelin receptor antagonists during EVLP may be useful for the rehabilitation of damaged donor lungs before transplantation.

## Data Availability

The datasets used and/or analysed during the current study are available from the corresponding author on reasonable request.
